# Absent Pericardium Causing Extreme Levoposition in a Child

**DOI:** 10.1002/ccr3.70047

**Published:** 2025-01-02

**Authors:** Filippos‐Paschalis Rorris, Alexandros Tsoutsinos, Meletios Kanakis

**Affiliations:** ^1^ Department of Pediatric and Adult Congenital Heart Surgery Onassis Cardiac Surgery Center Athens Greece; ^2^ Department of Pediatric Cardiology and Adult Congenital Heart Disease Onassis Cardiac Surgery Center Athens Greece

**Keywords:** absent pericardium, congenital heart disease, pericardial disease, sinus venosus

## Abstract

During surgical repair of congenital heart defects, surgeons often come across anomalies that might not have been adequately illustrated in preoperative imaging. In this case image, we describe the absence of pericardium which was discovered during surgical repair of a sinus venosus defect in a small child.

## 1 Case Presentation

**FIGURE 1:**
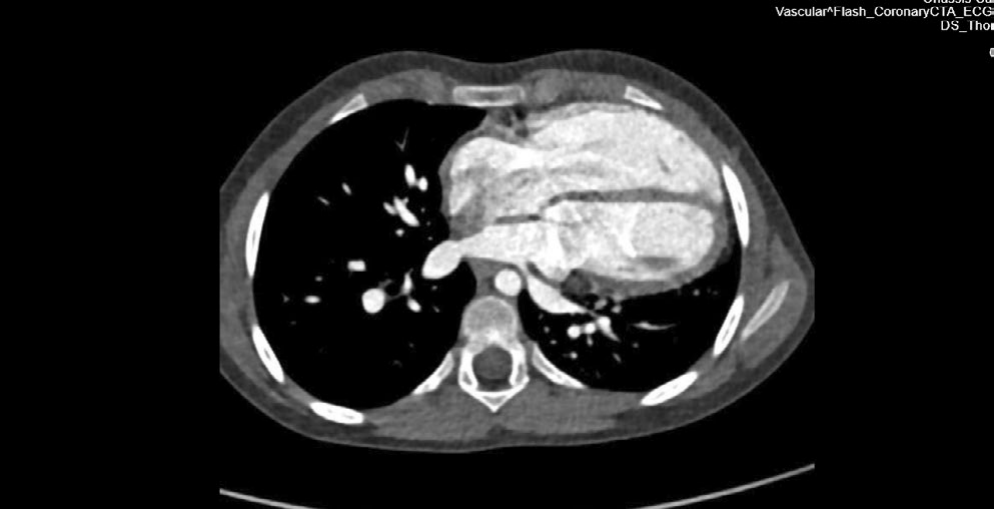
Preoperative computed tomography scan showing extreme levoposition.

**FIGURE 2:**
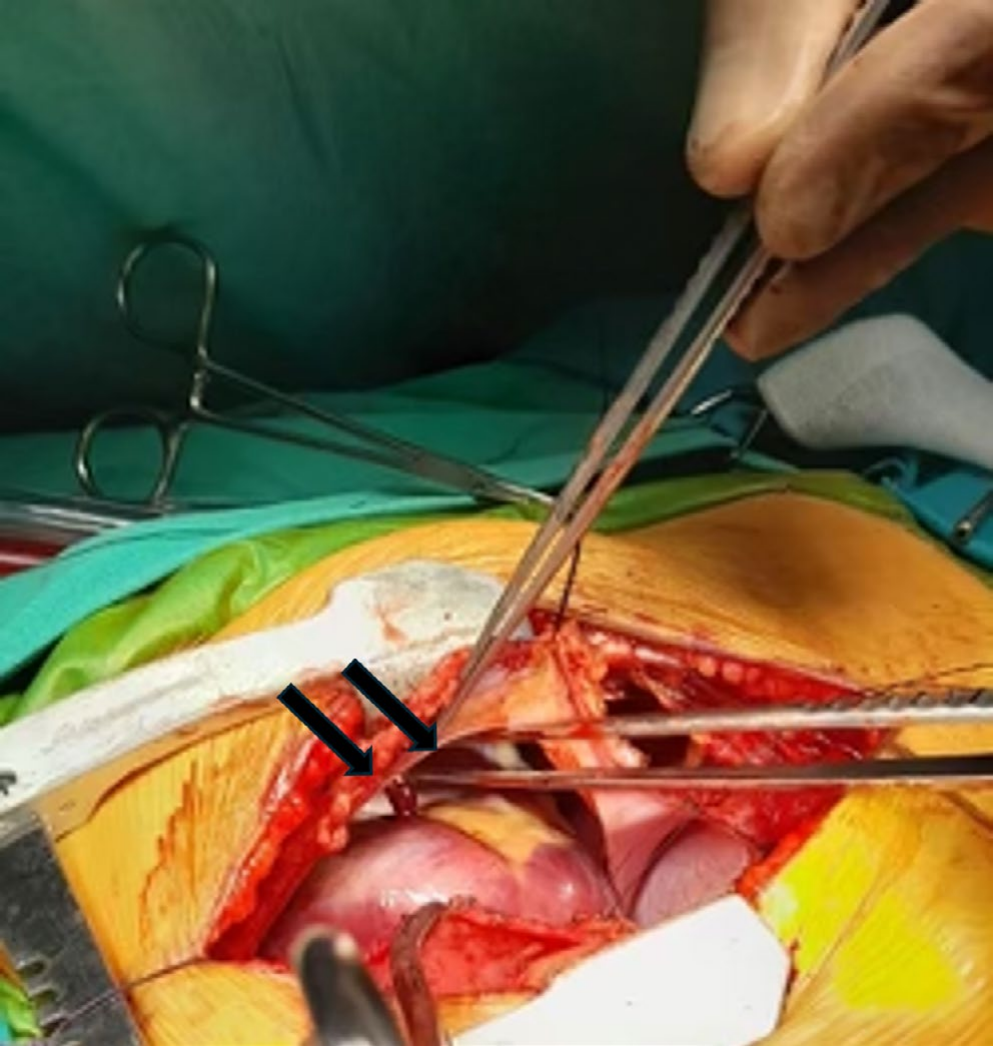
Intraopertive image showing the absence of the anterior portion the left pericardium (left phrenic nerve shown with tissue forceps).

A 6‐year‐old child was referred to our Pediatric Heart Surgery Department for surgical management of partial anomalous pulmonary vein connection and superior sinus venosus defect. The preoperative computed tomography scan (Figure 1) showed right heart chambers dilatation as well as extreme cardiac levoposition and leftward apical displacement. During the operation for sinus venosus surgical correction, we noticed the complete absence of the left anterior pericardial portion and partial absence of the anterior pericardial portion of the pericardial sac (arrows, Figure 2) which caused the heart to be displaced toward the left hemithorax. During this operation with the double patch technique, we normally harvest a large piece of autologous pericardium for reconstruction and repair of this congenital heart defect [1]. Due to the pericardial tissue absence, only a small piece of autologous pericardium was used as a patch for redirecting the anomalous pulmonary veins and repairing the sinus venosus defect. For the second patch, which is necessary for vessel enlargement of the superior vena cava, a piece of bovine pericardium was used. The child's postoperative course was uneventful.

Partial of complete absence of the pericardium are rare congenital disorders of the pericardial sac. Pericardial defects result after failure of complete fusion of the pleuropericardial membranes during the fourth week of embryonic life. The absence of pericardium is mostly asymptomatic and the defect is most commonly discovered during imaging or thoracic operations [2, 3]. There are four classifications of pericardial agenesis such as complete bilateral, complete left‐sided, complete right‐sided, and partial right‐ or left‐sided [2]. The most common type is the complete left‐sided with a reported prevalence of approximately 70% of cases. Partial right‐ or left‐sided agenesis are the rarest forms but are more likely to cause complications [2]. A simple chest X‐ray could be suggestive of the absent pericardium with the so‐called “snoopy sign.” However, the best imaging modality for diagnosis is cardiac magnetic resonance imaging [2]. In our case, the child had a preoperative echocardiogram and computed tomography angiography which had both missed the diagnosis of absent pericardium. Such anatomic anomaly is extremely rare and cardiac surgeons must be ready to adapt and change the planned operative course accordingly. The patient was last seen at follow‐up 3 months after the operation. The child is doing fine, has returned to normal activities, and has not been otherwise affected by the operation.

## Author Contributions

**Filippos‐Paschalis Rorris:** writing – original draft, image preparation. **Alexandros Tsoutsinos:** writing – original draft, writing – review and editing. **Meletios Kanakis:** supervision, writing – review and editing.

## Consent

Signed informed consent was obtained from the child's parents.

## Conflicts of Interest

The authors declare no conflicts of interest.

## Data Availability

Data sharing is not applicable to this article as no new data were created or analyzed in this study.

## References

[1] E. R. Kyger, 3rd , O. H. Frazier , D. A. Cooley , et al., “Sinus Venosus Atrial Septal Defect: Early and Late Results Following Closure in 109 Patients,” Annals of Thoracic Surgery 25, no. 1 (1978): 44–50. 619811 10.1016/s0003-4975(10)63485-6

[2] A. B. Shah and I. Kronzon , “Congenital Defects of the Pericardium: A Review,” European Heart Journal Cardiovascular Imaging 16, no. 8 (2015): 821–827. 26003149 10.1093/ehjci/jev119

[3] A. Balaji , R. Makam , N. Hussein , and M. Loubani , “Congenital Absence of Pericardium: A Case Report and Technical Considerations in Cardiac Surgery,” Cureus 16, no. 3 (2024): e56885 Published 2024 Mar 25. 38659528 10.7759/cureus.56885 PMC11041855

